# UAV Swarm Mission Planning in Dynamic Environment Using Consensus-Based Bundle Algorithm

**DOI:** 10.3390/s20082307

**Published:** 2020-04-17

**Authors:** Yaozhong Zhang, Wencheng Feng, Guoqing Shi, Frank Jiang, Morshed Chowdhury, Sai Ho Ling

**Affiliations:** 1 School of Electronics and Information, Northwestern Polytechnical University, Xi’an 710129, China; zhang_y_z@nwpu.edu.cn (Y.Z.); fengwencheng2018@mail.nwpu.edu.cn (W.F.); shiguoqing@nwpu.edu.cn (G.S.); 2 Center for Cyber Security Research and Innovation (CSRI), Deakin University, Geelong 3220, Australia; morshed.chowdhury@deakin.edu.au; 3 Centre for Health Technologies, School of Biomedical Engineering, University of Technology, Sydney 2007, Australia; steve.ling@uts.edu.au

**Keywords:** UAV, Sensors, Actuators, mission planning, improved CBBA

## Abstract

To solve the real-time complex mission-planning problem for Multiple heterogeneous Unmanned Aerial Vehicles (UAVs) in the dynamic environments, this paper addresses a new approach by effectively adapting the Consensus-Based Bundle Algorithms (CBBA) under the constraints of task timing, limited UAV resources, diverse types of tasks, dynamic addition of tasks, and real-time requirements. We introduce the dynamic task generation mechanism, which satisfied the task timing constraints. The tasks that require the cooperation of multiple UAVs are simplified into multiple sub-tasks to perform by a single UAV independently. We also introduce the asynchronous task allocation mechanism. This mechanism reduces the computational complexity of the algorithm and the communication time between UAVs. The partial task redistribution mechanism has been adopted for achieving the dynamic task allocation. The real-time performance of the algorithm is assured on the premise of optimal results. The feasibility and real-time performance of the algorithm are validated by conducting dynamic simulation experiments.

## 1. Introduction

The Unmanned Aerial Vehicles (UAVs) are widely used in the military battlefield nowadays, where the military practices are often challenged with more and more complex situations in contested tactic environments. With advanced sensors and precisive guidance weapons, a single UAV can basically perform a series of tasks such as investigation, attacking, and evaluation under complex situations. However, the ability of a single UAV to execute tasks is still limited. In order to accomplish the more sophisticated tasks in complex situations, multiple heterogeneous UAVs are usually adopted to perform cooperative operations. In multi-UAVs cooperative warfare, due to the different capabilities of UAVs, the diverse resources are needed to complete the tasks, and the time constraints between the tasks. Therefore, it is necessary to design a reasonable task planning scheme to attain the tasks and the sequential arrangement of the tasks. In battlefields, uncertainties of completion of tasks are an acute problem, for example, the new tasks short of UAV formation or the purge of UAV assigned to tasks often occur. Hence, the tasks reallocations in dynamic modes are needed. In addition, to achieve the real-time decision-making, it is necessary to reduce the communication loads between UAVs in the design phase of dynamic planning.

Task planning methods can be divided into two-fold, i.e., optimization methods and heuristic algorithms. Heuristic algorithms mainly include List Scheduling (LS) method, Clustering Algorithms [1] and Load Balancing (LB) method [2]. Intelligent optimization algorithm in nature is originated from the heuristic algorithm, which uses modern intelligent optimization algorithm to optimize the task planning for achieving the balance between the shortest solution time and the optimal solution. Its implementation is generally simple, and the solution quality is normally high. Intelligent optimization algorithms can be divided into the following categories: Evolutionary Algorithms (EA) [3,4], Swarm Intelligence Algorithms (SIA) [5,6], and other intelligent optimization algorithms [7,8,9]. The classification of task planning control system [10] is divided into three types: Centralized control, Distributed control, and Hierarchical distributed control. In the distributed sensory system, each agent acts as an independent decision-processing unit, which can communicate directly with each other. It has strong fault tolerance, flexibility and reliability [11,12,13,14].

Another example is the auction algorithm based on contract net [15] which is a distributed intelligent optimization algorithm. The process consists of four steps: bidding-bidding-bidding-winning. Each member in the processing stage makes independent decisions and connects through communications. Based on the contract network mechanism, Choi [16] proposes a Consensus-Based Auction Algorithm (CBAA) to solve the single task assignment problem. Meanwhile, a Consensus-Based Bundle Algorithms (CBBA) is developed further to solve the multi-task assignment problem. The feasibility of these two algorithms have been demonstrated in [17]. The CBAA and CBBA are both composed of task assignments and conflict mediations. Using these algorithms, a conflict-free optimal solution can be achieved. Since CBBA was proposed, therefore there is a scope to improve the algorithm to solve the more complex problem. Bertuccelli [18] improves CBBA by using Dijkstra algorithm, which solves the problems of path collision avoidance, and task allocation. These problems have been resolved in the environment of communication noise interference by setting the identical allocation with higher income. However, Bertuccelli did not consider all the environmental parameters to solve the complex problems, which is not suitable for real world situations. In [19], Choi extended his work by setting two different types of tasks in the simulation scenario and two corresponding heterogeneous UAVs, to improve the CBBA. Considering the huge traffic of CBBA in conflict mediation, Johnson [20] proposes an Asynchronous CBBA (ACBBA) algorithm, which uses a new set of local anti-collision rules that by passing the access of global information state to reduce the traffic. Mercker et al. also proposed an Extended version of CBBA (ECBBA) [21], where the authors considered task time constraints and able to reduce UAV traffic meaningfully. In [22], researchers Hunt et al. further improved CBBA to a Consensus Based Group Algorithms (CBGA). They set up each task to be resolved by multiple collaborated UAVs.

This method can improve 50% optimality of the solution but perform only single type of task. Smith et al. [23] improved the algorithm to Cluster-Formed Consensus-Based Bundle Algorithms (CFCBBA). By dividing UAV and tasks into several clusters, they are able to reduce overall computational costs, but their optimal solution couldn’t decrease the traffic [24,25]. Considering the emergence of new tasks in battlefields, Buckman etc. [26] proposes Partial Replanning algorithm called CBBA-PR. This algorithm takes part of the assigned tasks from the allocation state, and then mixes them with new tasks for reallocation purposes. They achieve the purpose of ensuring optimality and real-time performance in dynamic allocations, but the types of tasks allocated are single.

The contributions in this paper are three-fold: (1) We improve CBBA algorithm further by taking up multi-SEAD tasks as the main scenario, and considering the heterogeneity of UAV [27,28]. (2) We propose a new task planning scheme to solve the complex task of investigation-attack-evaluation. (3) To resolve the problem caused by the addition of new targets in the battlefield, the algorithm has been developed further to cope with the dynamic scenarios via minimizing the computation cost and communication of UAV. To ensure the optimal solution, we partially reconstructing the task set and asynchronous task assignment.

The structure of this paper is organized as follows: Section 2 presents the problem description and mathematical modeling; Section 3 discusses the task-planning algorithm; Section 4 simulates and validates the algorithm; Section 5 summarizes the work.

## 2. Problem Description and Mathematical Model

### 2.1. Problem Description

Suppression of Enemy Air Defense (SEAD) [29] refers to the combat activities of attacking enemy air defense systems in a specific area to make them temporarily or permanently incapacitated. Therefore, it weakens enemy air defense forces. In this paper, we refer the enemy air defense system is the enemy ground radar. The SEAD tasks for each enemy target include three sub-tasks: reconnaissance, attack, and evaluation. These three sub-tasks have strict timing requirements. In order to carry out the investigation and evaluation task, a UAV with corresponding sensors are needed to irradiate the target for a period. In terms of conducting the attack task, UAV needs to be equipped with arsenals, and the mission may require multiple of arsenals to hit the target and destroy it. The UAV has limited load capacity and can only mount a certain number of arsenals. The combat scenario is set as a two-dimensional area. At the beginning of the battle, there are a set of N Vehicles $V=\left\{V_{1},\ldots ,V_{N}\right\}$ and a set of M Targets $T=\left\{T_{1},\ldots ,T_{M}\right\}$. Each target $T_{j}$ contains three subtasks, i.e., Detect, Attack and Evaluate, $T_{j}=\left\{T_{j}^{D},T_{j}^{A},T_{j}^{E}\right\}$. The UAV will get benefits once completion of work of subtasks. However, all subtasks will not perform together in the same time as they may perform in the different time.

All UAVs carry sensors for reconnaissance and assessment missions.

To simplify the problem, the following assumptions are made.

- The energy shortage of sensors is not considered;
- The turning radius of UAV is not included;
- When the UAV is on mission in the area above the target, the speed is 0, the UAV hovers, and the rest of the time flies at a constant speed;
- Collision avoidance is ignored;
- The influence of duration is not considered.

### 2.2. Mathematical Model

In the process of task planning, due to the complexity of the task, a variety of constraints has been considered. The execution of the three subtasks of each target has strict timing requirements. The investigation task to be done first, followed by the strike task, and then the target is evaluated after the distraction of target. Set the time window $W_{j}\left(𝓽\right)$ for each subtask of target $T_{j}$ as follows:

(1) $$W_{j}\left(𝓽\right)=\{[{𝓽}_{j_{start}}^{D},{𝓽}_{j_{end}}^{D}],\{[{𝓽}_{j_{start}}^{A},{𝓽}_{j_{end}}^{A}],\{[{𝓽}_{j_{start}}^{E},{𝓽}_{j_{end}}^{E}]\},\;\forall j\in T$$

Among them, ${𝓽}_{j}^{D}$, ${𝓽}_{j}^{A}$, ${𝓽}_{j}^{E}$ represent the time window of target $T_{j}$ detection, attack and evaluation subtasks, start represents the start time of time window, and end represents the end time. The time constraints are as follows:

(2) $$0\leq {𝓽}_{j_{start}}^{D}\leq {𝓽}_{j_{end}}^{D}\leq {𝓽}_{j_{start}}^{A}\leq {𝓽}_{j_{end}}^{A}\leq {𝓽}_{j_{start}}^{E}\leq {𝓽}_{j_{end}}^{E}$$

In addition, the resource requirements of tasks and the resource carrying capacity of UAV should consider in mission planning. For investigation and evaluation of sub-tasks, only one UAV is needed for each task, and the execution time of UAV cannot be less than the shortest time required by the task. The constraints are as follows:

(3) $$\{\begin{array}{c} \sum_{i\in V}z_{k}^{i}\\ \underset{i\in V}{\sum }tc_{k}^{i}\geq tc_{k} \end{array},k\in \left\{T_{j}^{D},T_{j}^{E}\right\},\forall j\in T$$

The binary variable $z_{k}^{i}=1$ indicates that UAV $V_{i}$ performs subtasks k, $z_{k}^{i}=0$ indicates that UAV does not perform subtasks, $tc_{k}^{i}$ represents the time spent by UAV $V_{i}$ and performing subtasks k, $tc_{k}$ represents the time required to complete subtasks k.

For a strike mission, multiple arsenals are considered for each target to ensure its complete destruction. In addition, the total number of arsenals used by each UAV cannot exceed the total number of arsenals it carries. The resource constraints are as follows:

(4) $$\{\begin{array}{c} \underset{i\in V}{\sum }{𝓂}_{j}^{i}\cdot z_{j}^{i}\geq {𝓂}_{0}{}_{j}^{T},\forall j\in T\\ \underset{j\in T}{\sum }{𝓂}_{j}^{i}\cdot z_{j}^{i}\leq {𝓂}_{0}{}_{i}^{V},\forall i\in V \end{array}$$

Among them, ${𝓂}_{j}^{i}$ denotes the missile consumed by UAV $V_{i}$ when it strikes the target $T_{j}$; the binary variable $z_{j}^{i}=1$ denotes the UAV $V_{i}$ striking target $T_{j}$, and $z_{j}^{i}=0$ denotes the non-striking; ${𝓂}_{0}{}_{j}^{T}$ denotes the number of missiles needed to completely destroy the target $T_{j}$, and ${𝓂}_{0}{}_{i}^{V}$ denotes the initial number of missiles carried by UAV $V_{i}$.

In task planning, on the one hand, to maximize the total revenue, on the other hand, to ensure the shortest total mission time. We ensure the balance between the two parts when designing the revenue function. The revenue function consists of two parts: the revenue reward function and the distance discount function 11. Revenue reward function $R_{k}^{i}P_{i}$ represents the benefit of UAV $V_{i}$ and performing a specified subtask k along a predetermined path $P_{i}$. It is linked with two factors, one is the fixed benefit $r_{k}$ of UAV performing the subtask, the other one is the relationship between the time ${𝓽}_{arrival}$ when UAV arrives at the target area $T_{j}$ along the path $P_{i}$ and the time window $\left[{𝓽}_{k_{start}},{𝓽}_{k_{end}}\right]$ of subtask k. The larger the fixed income $r_{k}$ of subtask, the earlier the arrival time ${𝓽}_{arrival}$ and the higher the reward $R_{k}^{i}$.

(5) $$R_{k}^{i}P_{i}=r_{k}\cdot e^{-\lambda \left(\tau \left(P_{i}\right)-{𝓽}_{k_{start}}\right)}$$

Among them, λ is the scale factor, $\tau \left(P_{i}\right)$ indicates the time when UAV starts to execute subtask k along the path, $\tau \left(P_{i}\right)=\max\left({𝓽}_{arrival},{𝓽}_{k_{start}}\right)$ When $\tau \left(P_{i}\right)>{𝓽}_{k_{end}}$, the time to execute the task has been missed, $R_{k}^{i}(P_{i})=0$.

The distance discount function denotes the distance discount of UAV $V_{i}$ performing subtask k along the predetermined route $P_{i}$, which is associated to the total distance of UAV flying along the route. The longer the route, the greater the discount. By designing a reasonable distance discount function, the marginal revenue of UAV performing tasks along the path decreases. The flight distance of UAV $V_{i}$ arriving at subtask k along the pre-determined route $P_{i}$ is expressed by $d_{k}^{i}\left(P_{i}\right)$. There are the following calculation formulas:

(6) $$d_{k}^{i}\left(P_{i}\right)=d_{start}^{i}\left(P_{i}\right)+\underset{p\in P_{i}}{\sum }d_{p\to p+1}^{i}\left(P_{i}\right),\forall i\in V$$

(7) $$C_{k}^{i}P_{i}=\mu \cdot d_{k}^{i}\left(P_{i}\right)$$

where $d_{start}^{i}\left(P_{i}\right)$ represents the distance from the starting point to the first task point in the path, p represents each task point in the path, and $d_{p\to p+1}^{i}P_{i}$ represents the distance between two adjacent task points in the path. μ is the distance discount factor.

According to the above formulas, the task benefit function $S_{k}^{i}$ can be obtained as follows:

(8) $$S_{k}^{i}(P_{i})=\left(R_{k}^{i}(P_{i})-C_{k}^{i}(P_{i})\right)\cdot z_{k}^{i}$$

When the binary variable $z_{k}^{i}=1$, UAV $V_{i}$ is assigned to the task k, and when $z_{k}^{i}=0$, UAV is not assigned to the task. Thus, w the objective function f is obtained as follows:

(9) $$f=\underset{i\in V}{\sum }\underset{j\in T}{\sum }S_{k}^{i}(P_{i}),\;\forall k\in \left\{T_{j}^{D},T_{j}^{A},T_{j}^{E}\right\}$$

The constraints are shown in Equations (1)–(4).

## 3. Task Planning Algorithms

In our study, we adapt the CBBA based task-planning algorithm. This algorithm improves the tasks in multiple ways. For examples, maintaining strict sequence between tasks; improving the timing requirements of task assignment and able to perform well even agent carrying limited resources. The task assignment requires multi-agent collaboration whether it is a single task or multi-heterogeneous agent task assignment. The agent function and carrying resources are different. The real-time task assignment under dynamic conditions needs to dynamically add tasks, and ensure the real-time performance of the algorithm.

### 3.1. Introduction of CBBA

The CBBA algorithm is an auction algorithm based on contract network proposed by Choi [12]. The algorithm consists of two phases: (1) task selection and (2) conflict mediation. In the task selection phase, each agent tries to insert tasks into its own path set until all tasks are assigned or agent resources are exhausted to maximize the benefits of its own tasks. In the conflict mediation phase, the conflicts among the tasks assigned by each agent are eliminated, and the global total revenue is maximized. The two phases of task selection and conflict mediation are frequently circulated until the end of task assignment.

In CBBA algorithm, the task assignment and communication mediation among different agents are independent. Here each agent has certain information. They are:

$B_{i}=\left\{b_{i}^{1},b_{i}^{2},\ldots \right\}$: Task Bundle, which includes all tasks in battlefield known by agent $V_{i}$;
$P_{i}=\left\{p_{i}^{1},p_{i}^{2},\ldots \right\}$: Path set, representing all tasks assigned by agent $V_{i}$, is arranged in execution order;
$X_{i}=\left\{x_{i}^{1},x_{i}^{2},\ldots \right\}$: The highest bidder, where $x_{i}^{k}$ denotes agent $V_{i}$’s highest bidding agent for task $b_{i}^{k}$ in the task bundle $B_{i}$, and ∅ if no agent is bidding;
$Y_{i}=\left\{y_{i}^{1},y_{i}^{2},\ldots \right\}$: The highest bid, where $y_{i}^{k}$ represents the highest bid for task $b_{i}^{k}$ measured by agent $V_{i}$, and 0 if there is no bid;
$S_{i}=\left\{s_{i}^{1},s_{i}^{2},\ldots \right\}$: Timestamp, where $s_{i}^{j}$ denotes the time when agent $V_{i}$ received the last message from agent $V_{j}$ in the communication network;

### 3.2. Dynamic Task Generation

In the process of task assignment, we should consider not only the targets found before the simulation, but also the new targets in the battlefield. Each target generates a SEAD task, including three sub-tasks, detection, attack, and evaluation. These three subtasks have strict time constraints. Only after the pre-subtasks are completed, the post-subtasks can begin to execute. There is a coupling relationship between the time windows of the three subtasks. The task completion time of the pre-subtasks is the time window opening time of the post-subtasks. There is no special requirement for the time window width of the subtasks. Assuming that the target’s detection, attack, and evaluation subtasks are completed at ${𝓽}_{j_{finish}}^{D},{𝓽}_{j_{finish}}^{A},{𝓽}_{j_{finish}}^{E}$, respectively, the time window $W_{j}\left(𝓽\right)$ of each subtask of the target $T_{j}$ is:

$$W_{j}\left(𝓽\right)=\left\{\left[0,Inf\right],\left[{𝓽}_{j_{finish}}^{D},Inf\right],\left[{𝓽}_{j_{finish}}^{A},Inf\right]\right\},\forall j\in T$$

Due to the limitation of time window, each sub-task must be executed after the completion of its pre-task. Therefore, pre-task should be allocated before the allocation of each sub-task. After a sub-task assignment is completed, the order of tasks in the agent’s path will be determined, and the completion time of the sub-task will be resolute. Therefore, after the pre-task of a sub-task is assigned, its own time window will be determined. When assigning tasks according to the time constraints of subtasks, tasks can be managed in a way that generates tasks dynamically.

For each target, at the beginning of simulation, its detection subtasks can be executed. Thus, at the beginning of task assignment, the detection subtasks of each target are added to the task set of all agents. After a subtask is assigned, new subtasks will generate according to its type.

After assigning the reconnaissance sub-task, a new attack sub-task for the target $T_{j}$ is generated. The number of arsenals needed by the strike sub-task is ${𝓂}_{0}{}_{j}^{T}$, which destroys the target. However, due to the limited carrying capacity of the agent or the fact that the agent has already attacked other targets, the agent cannot destroy the target alone after bidding for this task. In that case, other agents are needed to assist. For this kind of scenario multi-agent requires attack task/sub -task to destroy target together. As the single agent cannot destroy target under sub-task therefore, single agent cannot get all the benefits of attacking the target.

Assuming that the number of arsenals required for the current attack mission k is ${𝓂}_{k_{need}}^{T}$, the number of remaining arsenals that the agent $V_{i}$ has not yet been assigned to other missions is ${𝓂}_{i_{last}}^{V}$, and the strike revenue of the target is c, the benefit of the attack mission can be calculated according to the following formula:

(10) $$S_{k}^{i}=\{\begin{array}{c} \frac{{𝓂}_{i_{last}}^{V}}{{𝓂}_{0}{}_{j}^{T}}\cdot c,\;\mathrm{if}\;{𝓂}_{i_{last}}^{V}\leq {𝓂}_{k_{need}}^{T}\\ \frac{{𝓂}_{k_{need}}^{T}}{{𝓂}_{0}{}_{j}^{T}}\cdot c,\;\mathrm{if}\;{𝓂}_{i_{last}}^{V}\geq {𝓂}_{k_{need}}^{T} \end{array}$$

As we mentioned in the previous para that target could not destroy by a single agent after assigning the strike task, then additional agents will continue the task. In this paper, we achieve this goal by generating new attack subtasks and assigning them. If ${𝓂}_{i_{last}}^{V}<{𝓂}_{k_{need}}^{T}$ indicates that the strike sub-task has not been completed, a new strike sub-task will be generated. The number of arsenals needed to complete the task is ${𝓂}_{k_{need}}^{T}-{𝓂}_{i_{last}}^{V}$ and the task time window remains unchanged. Then additional strike sub-task will add to the task set for all agents and assigned.

If the agent assigned to the sub-task has not completed thoroughly, a new sub-task will be generated and assigned until the sub-task is completed.

After the target attacking sub-task is completed, a new evaluation sub-task will be generated and added to the task set for all agents. Only one agent can complete each sub-task. The same type of targets and sub-tasks are added in the simulation process. First, generate detection sub-task followed by the strike sub-task and allocate it. Once the completion of the strike sub-task allocation occurs, the new evaluation sub-task will be generated and allocated.

The dynamic task generation algorithm is shown in Algorithm 1 below.

**Algorithm** **1.** Dynamic Task Generation.1:Insert the detect sub-tasks of each target into the task bundle of all agents2:**while** new subtasks can be generated3: **if** discovered new target $T_{j}$
4:  Insert the detect sub-tasks of target *j* into the task bundle of all agents5: **end if**8: **if** subtask *k* is assigned7:  **switch** type of subtask *k*8:   **case** detect: generating new attack subtasks; **break**9:   **case** attack: **if**
${𝓂}_{i_{last}}^{V}<{𝓂}_{k_{need}}^{T}$ generating new attack subtasks;10:     **else**: generating new evaluate subtasks; **break**11:   **case** evaluate: **break**12:  **end switch**13: **end if**14:
**end while**


### 3.3. Asynchronoous Task Alloction

In CBBA [11], in order to achieve a conflict-free optimal solution, all tasks in the task set need to be compared in the conflict mediation phase. In this way, the communication between agents is increased. To ease the communication traffic, a new communication mediation method has been proposed in this paper. Two drawbacks in the previous CBBA algorithm are identified in communications.

One is that at each point of time, all adjacent agents need to communicate with each other. However, multiple UAVs, which are not in conflict with other agents participating in the communication may increase network load. Another point is that in each communication, the pair of agents who do not handle certain tasks, but they need to exchange information of all tasks, which rises the traffic. This section will address these two points.

#### 3.3.1. Task Selection

Firstly, the state of tasks in task bundle is divided into the following four categories: auction, assigned, executing and completed. Among them, the auction indicates that the task is in the bidding state, and the agent can participate in the bidding of the task. The “assigned” indicates that the task has been assigned to the current agent. The “executing” indicates that an agent is performing the task and the “completed” indicates that the task has been completed. When the status of a task is in executing or completed types, the agent that performs the task sends the status information of the task to other agents through the communication network. And the agent that receives the message changes the status of the task in its own task set. In each task selection process, only one task is added to the agent’s path set $P_{i}$, and this task is the only object of this communication mediation. The task selection algorithm is illustrated in Algorithm 2.

**Algorithm** **2.** Task Selection.1:Get a set $B_{i}^{a}$ of all tasks that are auctioned2:
**for**
$k\in B_{i}^{a}$
3: $S_{k}^{i}\left(P_{i}\right)=\max_{n\leq \left|P_{i}\right|+1}S_{k}^{i}\left(P_{i}{⨁}_{n}\left\{k\right\}\right),\forall k\in B_{i}^{a}$4: $h_{i,k}=\{\begin{array}{l} 1,\mathrm{if}\;S_{k}^{i}\left(P_{i}\right)>y_{i}^{k}\\ 0,\mathrm{if}\;S_{k}^{i}\left(P_{i}\right)\leq y_{i}^{k} \end{array}$5:
**end for**
6:
$S_{k^{*}}^{i}\left(P_{i}\right)=\max_{k\in B_{i}^{a}}S_{k}^{i}\left(P_{i}\right)\cdot h_{i,k}$
7:
$k^{*}=\arg\max_{k\in B_{i}^{a}}S_{k}^{i}\left(P_{i}\right)\cdot h_{i,k}$
8:
$n_{i,k^{*}}=\arg\max_{n}S_{k^{*}}^{i}\left(P_{i}{⨁}_{n}\left\{k^{*}\right\}\right)$
9:
**if**
$S_{k^{*}}^{i}\left(P_{i}\right)>0$
10: Change the state of task $k^{*}$ to assigned in $B_{i}$11: $x_{i}^{k^{*}}=i$, $y_{i}^{k^{*}}=S_{k^{*}}^{i}\left(P_{i}\right)$12: $P_{i}=P_{i}{⨁}_{n_{i,k^{*}}}\left\{k^{*}\right\}$
 **if** the state of task $k^{*}$ in $B_{i}$ is attack
  ${𝓂}_{k^{*}{}_{consume}}^{T}=\{\begin{array}{ll} {𝓂}_{i_{last}}^{V}, & \mathrm{if}\;{𝓂}_{i_{last}}^{V}\leq {𝓂}_{k^{*}{}_{need}}^{T}\\ {𝓂}_{k^{*}{}_{need}}^{T}, & \mathrm{if}\;{𝓂}_{i_{last}}^{V}>{𝓂}_{k^{*}{}_{need}}^{T} \end{array}$
  ${𝓂}_{i_{last}}^{V}={𝓂}_{i_{last}}^{V}-{𝓂}_{k^{*}{}_{need}}^{T}$
 **end if**13:
**end if**


When adding tasks to the path set $P_{i}$, we first find the set $B_{i}^{a}$ of tasks whose states are auction in the task set $B_{i}$. For each task k, we try to insert them into the path set so that the task can get the highest revenue. The score $S_{k}^{i}\left(P_{i}{⨁}_{n}\left\{k\right\}\right)$ of the nth position in the task insertion path set $P_{i}$ can be calculated by Formula (9). Trying to insert task k into each location of the path set, the maximum score obtained is the score $S_{k}^{i}\left(P_{i}\right)$ of agent $V_{i}$ executing task k. Here the score calculation formula is as follows:

(11) $$S_{k}^{i}\left(P_{i}\right)=\max_{n\leq \left|P_{i}\right|+1}S_{k}^{i}\left(P_{i}{⨁}_{n}\left\{k\right\}\right),\forall k\in B_{i}^{a}$$

Comparing the score $S_{k}^{i}\left(P_{i}\right)$ with agent $V_{i}$’s highest bid $y_{i}^{k}$ for task k, the binary variable $h_{i,k}$ is obtained as follows:

(12) $$h_{i,k}=\{\begin{array}{l} 1,\;\mathrm{if}\;S_{k}^{i}\left(P_{i}\right)>y_{i}^{k}\\ 0,\;\mathrm{if}\;S_{k}^{i}\left(P_{i}\right)\leq y_{i}^{k} \end{array}$$

Then the maximum score of each task is compared to find the highest score $S_{k^{*}}^{i}\left(P_{i}\right)$ and the task corresponding $k^{*}$ and the best insertion position $n_{i,k^{*}}$. The formula is as follows:

(13) $$S_{k^{*}}^{i}\left(P_{i}\right)=\max_{k\in B_{i}^{a}}S_{k}^{i}\left(P_{i}\right)\cdot h_{i,k}$$

(14) $$k^{*}=\arg\max_{k\in B_{i}^{a}}S_{k}^{i}\left(P_{i}\right)\cdot h_{i,k}$$

(15) $$n_{i,k^{*}}=\arg\max_{n}S_{k^{*}}^{i}\left(P_{i}{⨁}_{n}\left\{k^{*}\right\}\right)$$

If $S_{k^{*}}^{i}\left(P_{i}\right)>0$ indicates that the agent $V_{i}$ has bid for the task $k^{*}$, then the state of the task will be changed to assigned in the task set $B_{i}$, the highest bid will be changed to $S_{k^{*}}^{i}\left(P_{i}\right)$, and the highest bidder will be changed to agent. The task $k^{*}$ is then inserted at the $n_{i,k^{*}}$th position in the path set $P_{i}$. The information update formula is as follows:

(16) $$x_{i}^{k^{*}}=i,y_{i}^{k^{*}}=S_{k^{*}}^{i}\left(P_{i}\right)$$

(17) $$P_{i}=P_{i}{⨁}_{n_{i,k^{*}}}\left\{k^{*}\right\}$$

If the task $k^{*}$ is a strike mission, after inserting it into the path set, then arsenals requirements ${𝓂}_{k^{*}{}_{need}}^{T}$ of the mission and the arsenal surplus ${𝓂}_{i_{last}}^{V}$ of the agent, the arsenal consumption ${𝓂}_{i_{last}}^{V}$ of the mission will adjust as follows, and the following changes are made to the remaining arsenal of the agent:

(18) $${𝓂}_{k^{*}{}_{consume}}^{T}=\{\begin{array}{ll} {𝓂}_{i_{last}}^{V}, & \mathrm{if}\;{𝓽}_{i_{last}}^{V}\leq {𝓂}_{k^{*}{}_{need}}^{T}\\ {𝓂}_{k^{*}{}_{need}}^{T}, & \mathrm{if}\;{𝓂}_{i_{last}}^{V}>{𝓂}_{k^{*}{}_{need}}^{T} \end{array}$$

(19) $${𝓂}_{i_{last}}^{V}={𝓂}_{i_{last}}^{V}-{𝓂}_{k^{*}{}_{need}}^{T}$$

#### 3.3.2. Conflict Mediation

When agent $V_{i}$ is assigned to a new task, unlike the previous CBBA synchronous communication mode, the agent does not need to wait for a specific time point. In this paper, an asynchronous communication method is used, which sends the information of the task to the agent who communicates directly with it after it is assigned to the task, without waiting. If the receiver changes its own information according to the sending information, the updated information about the task will send the agent directly. The task information sent by the agent includes the highest bid and the corresponding bidder. By means of asynchronous communication and sending only single task information, the traffic can be reduced significantly. The objective of conflict mediation is to ensure that each sub-task can only be assigned maximum of one agent.

After receiving the information about task k from agent $V_{i}$, agent $V_{j}$ can take three actions: update, reset and leave. Specifically, as follows:

- update: $x_{j}^{k}=x_{i}^{k}$, $y_{j}^{k}=y_{i}^{k}$, $B_{k_{status}}^{j}=auction$;
- reset: $x_{j}^{k}=\emptyset$, $y_{j}^{k}=0$, $B_{k_{status}}^{j}=auction$;
- leave: $x_{j}^{k}=x_{j}^{k}$, $y_{j}^{k}=y_{j}^{k}$, $B_{k_{status}}^{j}=B_{k_{status}}^{j}$.

Where $B_{k_{status}}^{j}$ denotes the state of task k considered by agent $V_{j}$ in task set $B_{j}$.

The conflict mediation rules are shown in Table 1, where the first column represents the highest bidder that the sender agent $V_{i}$ reflects about task k, the second column is the highest bidder that the receiver agent $V_{j}$, and the third column is the action taken by the agent $V_{j}$.

After the agent $V_{j}$ acts on task k according to the rules provided in Table 1, it compares with the task status before the agent acts. If the state of task k is assigned in the task set $B_{j}$, before the action commences. Then it indicates that the state of task k has changed. Therefore, task set and path set of the agent should update and delete the task k. In addition, if the task is a strike task, then the agent’s surplus arsenal will be restored. The arsenal residual update formula is as follows:

(20) $${𝓂}_{j_{last}}^{V}={𝓂}_{j_{last}}^{V}+{𝓂}_{k_{consume}}^{T}$$

#### 3.3.3. Offline Task Assignment

The methods of task dynamic generation, task selection and conflict mediation have been introduced. In the current CBBA has two steps of task selection and conflict mediation, through the continuous cycle of two phases. Then a conflict-free optimal solution is sought.

As the current form of CBBA algorithm is not capable to address the very complex tasks, because each target contains multiple subtasks with strict timing constraints, and some subtasks required multiple agents to complete the tasks. Therefore, in this paper, the task dynamic generation method is introduced to simplify the complex tasks.

Offline task assignment refers to the assignment of all existing tasks before the agent starts to perform the tasks. At this point, state of agents and tasks numbers are fixed and do not change with the environment. The offline task assignment algorithm is shown in Algorithm 3:

**Algorithm** **3.** Offline Task Assignment.1:
*Dynamic Task Generation*
2:**while** some tasks have not been assigned3: ***phase 1***: *Task selection* and the currently assigned task is *k*4: ***phase 2***: *Conflict mediation* for task *k*5: ***phase 3***: *Dynamic task generation* based on task *k*6:
**end while**


The offline task assignment algorithm has three phases: (1) task dynamic generation, (2) task selection, and (3) conflict mediation. The three phases form a loop in order until the assignment ends. First, at the beginning of the task assignment, the algorithm 1 is used to generate the detection subtasks for each target. Then insert them into the task set of all agents and start the loop to perform task assignment. In the task assignment process, each agent makes independent decisions in each task selection phase, and each cycle only adds a new task to the path set. If an agent is assigned to a new task, the task information is sent immediately to other agents for conflict mediation. In the conflict mediation phase, assign the currently processed subtask to one agent. After the conflict mediation, enter the task dynamic generation phase, generate a new subtask according to the currently assigned subtask, and then enter the task selection phase again to perform a new round of loop. At the end of the loop, all tasks in each agent’s task set have their highest bidder, which enables the agent to think that all tasks are assigned, and they have performed.

#### 3.3.4. Dynamic Task Assignment

During the simulation process, a new target $T_{new}$ may be encountered, and new tasks brought by the new targets need to be assigned to the agents. In traditional dynamic task allocation, the original path set of agents is deleted; and all tasks are released with new mixed tasks. Then reallocated the tasks. This allocation method incurred a large amount of computational cost, and it is difficult to guarantee the real-time performance of the algorithm in the rapidly changing battlefield. In this paper, the task allocation method of path set is reconstructed partially and adopted. Only releasing the allocation state of some low-revenue tasks and then reallocating can seek the optimal allocation scheme sought while ensuring the real-time of the solution. The dynamic task assignment algorithm is outlined in Algorithm 4.

**Algorithm** **4**. Dynamic Task Assignment1:**if** new target $T_{new}$ was discovered2: **for** all $V_{i}$ in V
3:  $B_{i}^{aa}=\emptyset$4:   **for** all task k in $B_{i}$
5:    **if** task *k* is a detection task and the status is auction or assigned.6:     $B_{i}^{aa}=B_{i}^{aa}\cup k$7:    **end if**8:   **end for**
9:   **for** all target $T_{j}$ hasn’t been executed10:    $S_{j}=\underset{k\in B_{i}^{aa}}{\sum }y_{i}^{k}\cdot h_{k}$ # Gain total revenue of $T_{j}$
11:    $h_{k}=\{\begin{array}{l} 1,\mathrm{if}\;target_{k}=j\\ 0,\mathrm{if}\;target_{k}\neq j \end{array}$12:   **end for**13:   $T^{\prime }=\emptyset \cup T_{j},\forall S_{j}\in \min_{\left|S\right|\cdot \varepsilon }S$14:   ${𝓂}_{i_{last}}^{V}={𝓂}_{i_{last}}^{V}+\underset{k\in P_{i}}{\sum }{𝓂}_{k_{consume}}^{T}\cdot T_{k}$15:   $B_{i}=B_{i}⊖b_{i}^{k};\forall target_{k}\in T^{\prime }$16:   $P_{i}=P_{i}⊖p_{i}^{k};\forall target_{k}\in T^{\prime }$17:   $T^{\prime }=T^{\prime }\cup T_{new}$18: **end for**19: **Offline Task Assignment**20:
**end if**


After the discovery of a new target, the assignment status of some tasks is removed first, and then the new tasks are reallocated together. To do that, a distributed control method is proposed and implemented. In this method, each agent performs independently through task distribution scheme.

For the agent $V_{i}$, in order to ensure that the tasks being executed or completed are not affected, as long as all the targets corresponding to the auction or assigned subtasks are found, then the task set $B_{i}^{aa}$ related to these targets are found in the task set $B_{i}$, and then calculate the total revenue $S=\left\{S_{1},S_{2},\ldots \right\}$ of these targets according to the Equation (21).

(21) $$S_{j}=\sum_{k\in B_{i}^{aa}}y_{i}^{k}\cdot h_{k}$$

(22) $$h_{k}=\{\begin{array}{l} 1,\;\mathrm{if}\;target_{k}=j\\ 0,\;\mathrm{if}\;target_{k}\neq j \end{array}$$

where $target_{k}$ represents the target corresponding to task k, and then a set $T^{\prime }$ of partial targets with the lowest total revenue is obtained according to Equation (23):

(23) $$T^{\prime }=\emptyset \cup T_{j},\forall S_{j}\in min_{\left|S\right|\cdot \varepsilon }$$

Among them, |S| denotes the total number of targets that have not yet been executed, and ε∈[0,1] is a proportional factor, indicating the proportion of the target number to |S| for reallocation. If ε is larger then more reallocated targets are, and the closer to the optimal solution. The smaller ε is, the smaller the number of reallocated targets, the higher the reallocation efficiency then $\min_{\left|S\right|\cdot \varepsilon }S$ represents the targets of the smallest total revenue.

After that, each task k in the path set $P_{i}$ is judged. If the task corresponding to the target belongs to $T^{\prime }$ and is a strike task, then arsenal surplus of agent $V_{i}$ needs to be updated:

(24) $${𝓂}_{i_{last}}^{V}={𝓂}_{i_{last}}^{V}+\sum_{k\in P_{i}}{𝓂}_{k_{consume}}^{T}\cdot T_{k}$$

The binary variable $T_{k}=1$ indicates the target corresponding to task k belongs to $T^{\prime }$ and is a strike task, and $T_{k}=0$ indicates that the above two conditions are not satisfied.

In addition, all tasks corresponding to the target $T^{\prime }$ need to be deleted in the task set $B_{i}$ and the path set $P_{i}$:

(25) $$B_{i}=B_{i}⊖b_{i}^{k},\forall target_{k}\in T^{\prime }$$

(26) $$P_{i}=P_{i}⊖p_{i}^{k},\forall target_{k}\in T^{\prime }$$

where ⊖ means to remove the task from the set.

Then merge the new target $T_{new}$ into the set $T^{\prime }$ according to Equation (27):

(27) $$T^{\prime }=T^{\prime }\cup T_{new}$$

Finally, the tasks generated by the targets in the set $T^{\prime }$ are treated as new tasks, and the offline task allocation algorithm is used for allocation.

## 4. Simulation

To verify the feasibility of the improved CBBA to solve the complex task allocation and the real-time performance of task allocation, simulation experiments are designed to verify it. The simulation uses QT5.12.2 platform, the programming language is C++, the computer processor is Intel (R) Core (TM) i7-8700 CPU@3.20GHZ, the memory is 16.0 GB, and the system is Windows 10 Professional.

The simulation performance is depicted in three simulation scenarios, which are multi-UAV offline reconnaissance task assignment, heterogeneous UAV offline complex task assignment, and heterogeneous UAV real-time complex task assignment. The simulation results include: (1) UAV task route, showing the results of UAV task allocation and action route; (2) UAV task time, showing the time each UAV stays at the task point, and proving timing constraints between tasks; (3) algorithm calculation time, showing the time spent in algorithm calculation at each moment during the simulation, and showing the real-time performance of the algorithm.

### 4.1. Multi-UAV Offline Reconnaissance Task Assignment

#### 4.1.1. Simulation Scene

In a 2-D simulation area, a total of 8 UAVs and 50 targets are included. The UAV needs to complete the reconnaissance task for all targets, and each target’s reconnaissance task can be completed with only one UAV. Each UAV carries sufficient reconnaissance load and is capable of detecting all targets. During the reconnaissance of each target, it takes 5 s to stay above the target in order to obtain sufficient target information. At the initial moment, UAV and targets are randomly distributed in a 25 km × 15 km rectangular area. All targets are fixed targets, and they are added before the simulation starts. At the beginning of the simulation, each UAV performs task assignment independently and resolves conflicts through communication. After the task assignment is completed, the UAV starts to execute tasks in order. After the UAV starts to operate, the task assignment is no longer performed.

#### 4.1.2. Simulation Result

**Figure 1:**
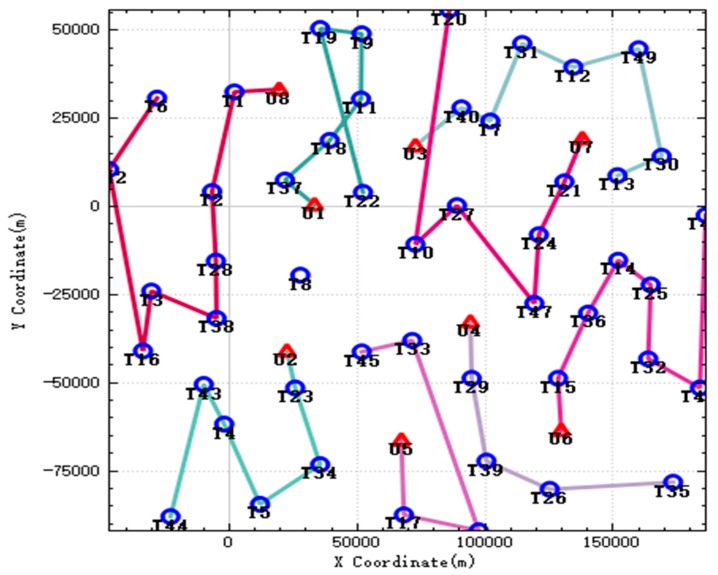
UAV mission route.

The sequence of UAVs performing tasks is shown in Figure 1.

In the figure, the red triangle symbol represents the initial position of the UAV, and the blue circle symbol represents the position of the target. It can be seen from the figure that UAV has performed all tasks, and each target is only detected by one UAV, and there is no task conflict. The number of UAV tasks is close, indicating that the algorithm balances the number of tasks for each UAV. When UAV executes the assigned tasks, its route is also optimal, ensuring the shortest task time.

**Figure 2:**
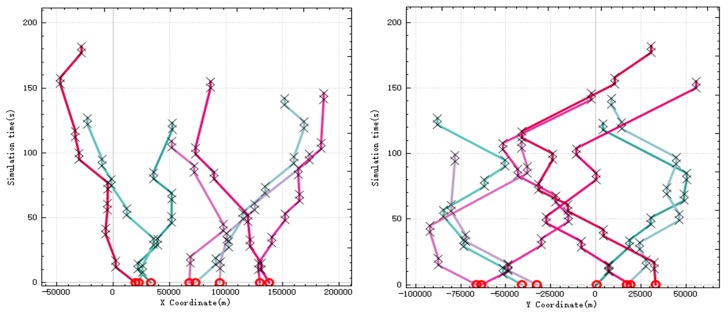
UAV position over time.

When UAV performs reconnaissance missions, it needs to use sensors to continuously irradiate the target for a period of time, so that UAV will stay above the target for a period of time. In Figure 2, the left figure is the relationship between *X* coordinate and time of UAV, and the right figure is the relationship between *Y* coordinate and time. In the figure, the circle symbol represents the initial position of the UAV, and the two black crosses are in a group, representing the start time and end time when the UAV is detecting a target. It can be seen from the figure that UAV stayed at each target for enough time to detect it.

**Figure 3:**
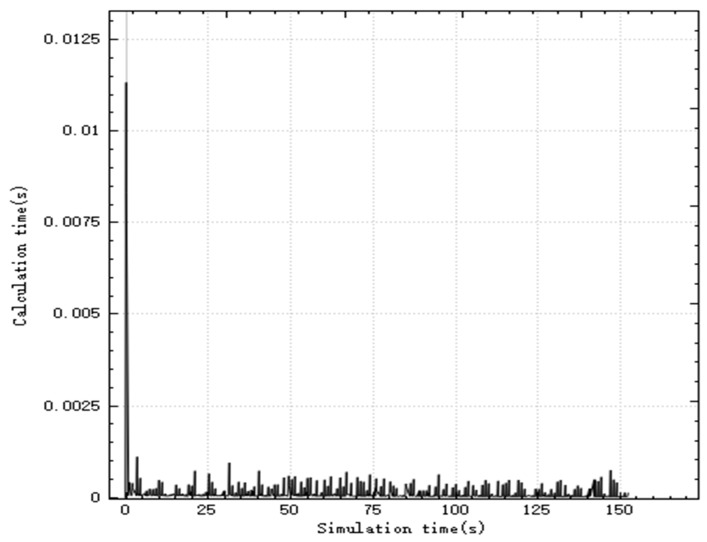
Calculate time-consuming curve in each step.

In the improved CBBA, asynchronous task allocation is used to distribute the calculation of the entire allocation process. Figure 3 shows the time consumed by the algorithm calculation thread in each step during the simulation. The simulation step is 0.03 s, and the peak time for a single step calculation is 0.011 s, which is less than the simulation step. The total task allocation ends in 0.5 s, and a global task allocation scheme is quickly given to ensure real-time performance.

### 4.2. Heterogeneous UAV Offline Complex Task Assignment

#### 4.2.1. Simulation Scene

In the offline complex task assignment scenario of heterogeneous UAVs, all targets include a SEAD task, consisting of three subtasks: reconnaissance-strike-evaluation, and three subtasks have timing constraints. When performing reconnaissance and evaluation missions, UAVs need to carry sensors to irradiate them for a period of time to obtain target information. When performing strike missions, UAVs need to fire certain weapons to destroy them. The simulation scene contains a total of 30 UAVs and 40 targets. Each UAV carries 4 weapons. Each target requires 3 weapons to be completely destroyed. The target requires 5 s for reconnaissance, 2 s for strike, and 5 s for evaluation. Before the simulation, all UAVs and targets have been added to the battlefield, and the locations are randomly distributed in a 2-dimensional area. All targets are fixed targets and do not move with time.

#### 4.2.2. Simulation Result

**Figure 4:**
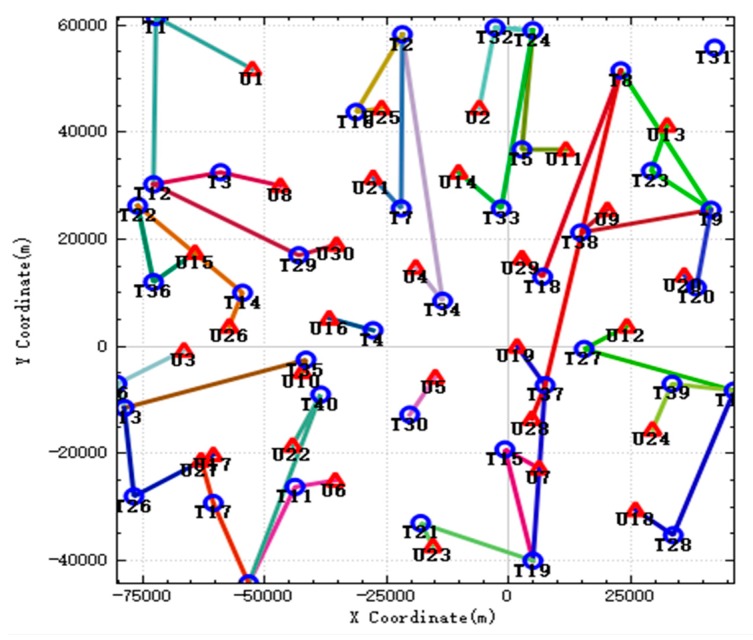
The result of UAV task allocation.

In the improved CBBA algorithm, the tasks that meet the timing constraints are assigned to each UAV strictly according to the timing by using the method of dynamically generating subtasks, and the multiple UAV tasks are disassembled into multiple subtasks and assigned to multiple UAVs so that they can work together. The results and execution route of the UAV task assignment are shown in Figure 4. When the UAV strikes some targets, because the UAV has consumed some ammunition when completing the previous strike mission, there is not enough ammunition to destroy the current target. Therefore, when performing these tasks, multiple UAVs need to work together to complete their strike mission. In the picture, targets such as T10 and T13 are attacked by two UAVs simultaneously. Therefore, when performing these tasks, multiple UAVs are needed to complete them. In the figure, targets such as T10 and T13 are simultaneously attacked by two UAVs.

In the improved CBBA, the tasks that meet the timing constraints are assigned to each UAV strictly according to the timing by using the method of dynamically generating subtasks, and the multiple UAV tasks are disassembled into multiple subtasks and assigned to multiple UAVs so that they can work together. The results and execution route of the UAV task assignment are shown in Figure 4. When the UAV strikes some targets, because the UAV has consumed some ammunition when completing the previous strike mission, there is not enough ammunition to destroy the current target. Therefore, when performing these tasks, multiple UAVs need to work together to complete their strike mission. In the picture, targets such as T10 and T13 are attacked by two UAVs simultaneously. Therefore, when performing these tasks, multiple UAVs are needed to complete them. In the figure, targets such as T10 and T13 are simultaneously attacked by two UAVs.

**Figure 5:**
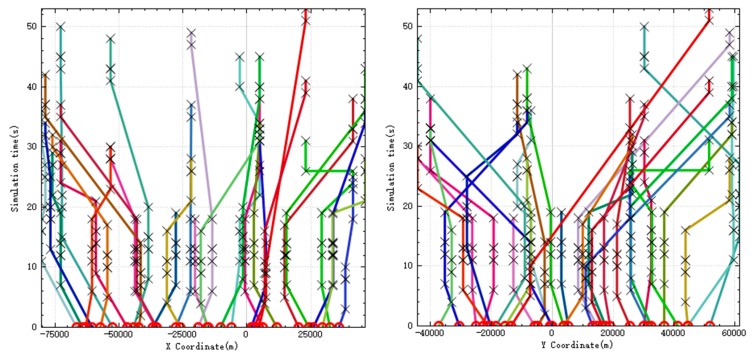
UAV position over time.

Because UAVs have timing constraints when they perform reconnaissance, strike, and evaluation tasks, they cannot begin “execution” until the pre-mission tasks are completed. The curve of UAV position over time is shown in Figure 5. The left figure shows the relationship between *X* coordinate and time, and the right figure shows the relationship between Y coordinate and time. In the figure, every two crosses are a group, which represents the start time and end time of the subtask. It can be seen from the figure that the mission time of each target completed by a single UAV is divided into 3 segments, from bottom to top: reconnaissance, strike, and evaluation. For the target that needs to be attacked by two UAVs, the mission time is divided into four sections. After the first UAV completes its reconnaissance and the partial task of strike, the second UAV completes its remaining strike task and evaluates the target after the target is destroyed. As you can see, all tasks are performed in sequence.

**Figure 6:**
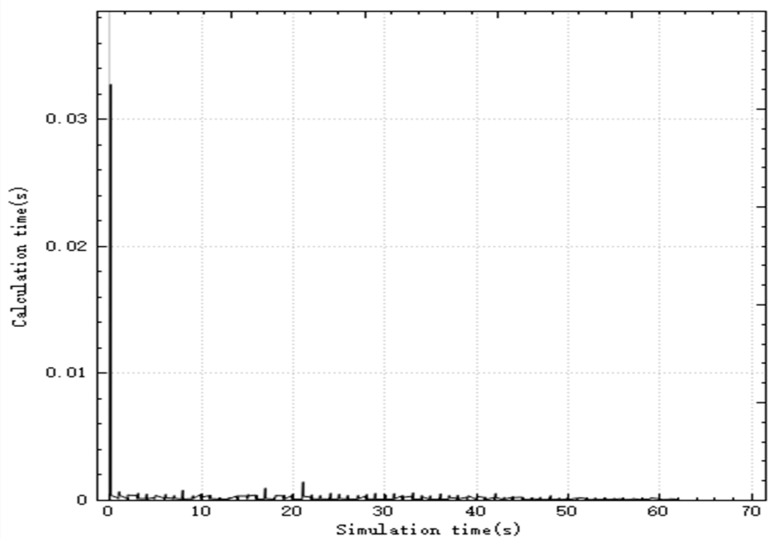
Calculate time-consuming curve in each step.

Figure 6 is a graph showing the variation of the calculation time of the improved CBBA in the simulation with the simulation time. It can be seen from the figure that the peak time of the single-step calculation of the simulation is 0.033 s, which meets the real-time requirements.

### 4.3. Heterogeneous UAV Real-time Complex Task Assignment

#### 4.3.1. Simulation Scene

Before the simulation began, there were a total of 8 UAVs on the battlefield, each UAV carrying sensors and 9 weapons. In addition, 32 enemy targets have been found on the battlefield, and each target contains a SEAD mission, which needs to be reconnaissance, attacked and evaluated.

Each target requires 2 weapons to be destroyed. The target’s reconnaissance takes 5 s, the strike takes 2 s, and the evaluation takes 5 s. After the simulation starts, new targets will continue to appear. These targets are of the same type as the previous targets and include a SEAD task.

After adding new targets, each UAV redistributes its tasks.

#### 4.3.2. Simulation Result

**Figure 7:**
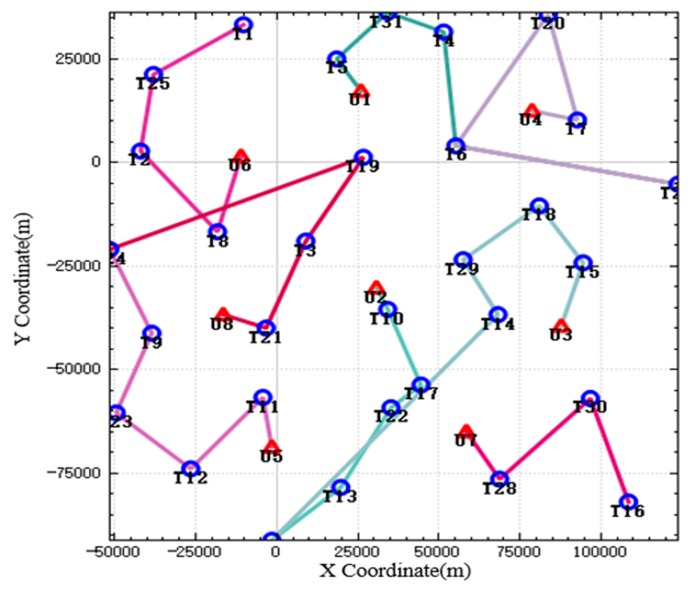
Allocation results before and after the new target appears.

After the simulation begins, new targets will appear in the battlefield, and the UAVs need to redistribute and execute the tasks brought by these new targets. In the Figure 7, the left figure shows the task assignment results before the emergence of the new target. As can be seen from the figure, due to the sufficient load of weapons carried, the UAV can complete most of the tasks alone, and all tasks are assigned and executed. The right figure shows the task assignment results after the new target appears. The black circle marks in the figure represents the new targets. Compared with the previous ones, the task assignment results have changed a lot. A total of 4 new targets appeared during the simulation.

Because the UAV has already consumed a lot of ammunition when performing previous tasks, and there are not enough weapons to strike the new targets, more tasks need to be completed by multiple UAVs.

**Figure 8:**
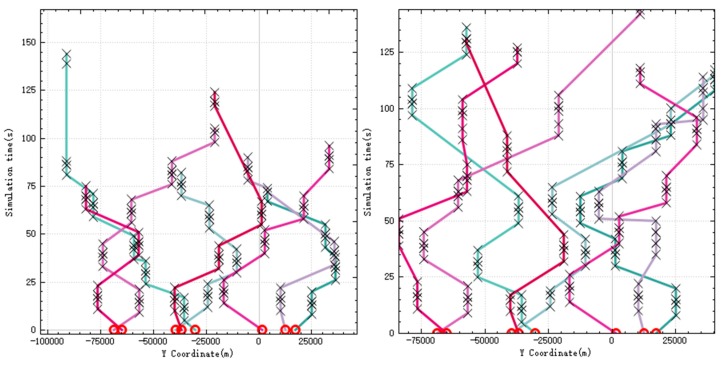
UAVs’ *Y* coordinate over time.

The new targets appearing on the battlefield are the same as the types of targets that previously existed, and the reconnaissance, strike, and evaluation tasks they produce also have timing constraints. Figure 8 shows the relationship between the *Y* coordinate and time of the UAV during the simulation. The left figure shows the relationship before the new target appears, and the right figure shows the relationship after the occurrence. It can be seen from the figure that before and after the new target appears, the task allocation results meet the requirements of timing constraints.

**Figure 9:**
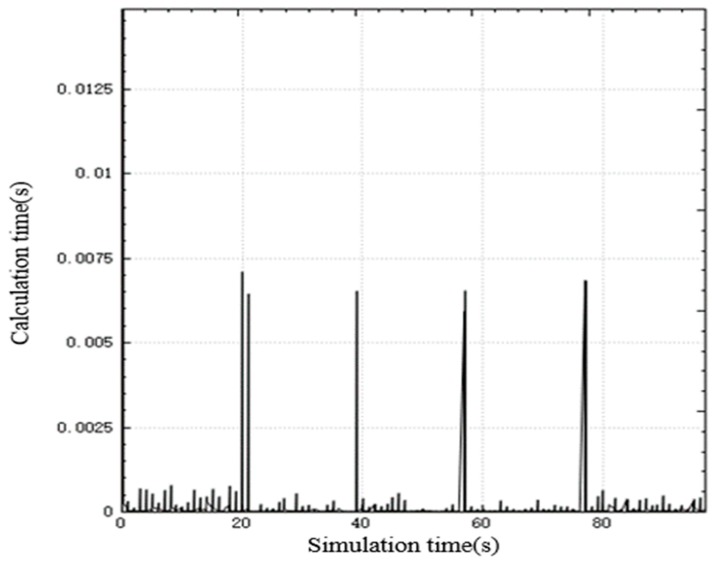
Calculate time-consuming curve in each step.

Figure 9 shows the relationship between the calculation time and simulation time of the algorithm. As can be seen from the figure, the algorithm has the largest calculation amount when starting off-line task allocation, with a peak time consumption of 0.0125 s, and the calculation time of the algorithm after that is about 0 ss. After each new target appears in the battlefield, UAV performs rapid task redistribution. By reconstructing part of the task bundle, the peak time of real-time task assignment calculation does not exceed 0.0075 s, which meets the algorithm’s real-time performance requirements.

**Figure 10:**
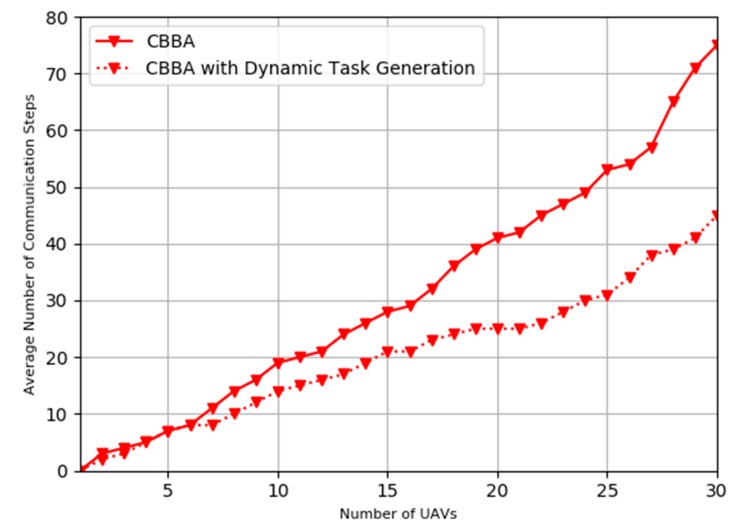
Comparison of average number of communication steps for consensus.

The further experiment of the performance comparison has been conducted against the conventional CBBA algorithm, our improved CBBA algorithm with the dynamic task generation/allocation process outperform the conventional CBBA, Figure 10 shows our proposed scheme takes less average number of communication steps for consensus in various swarm sizes when the emergent missions occur. We can see the extended CBBA approach exhibits a faster response with less steps.

## 5. Conclusions

This paper investigates the real-time complex task planning problem of Multi-heterogeneous UAVs in dynamic and uncertain environments. This work considers the following constraints for the task allocation: with time sequence constraints; under resource constraints; task allocation that requires multi-UAVs to cooperate; task allocation of Multi-heterogeneous UAVs as well as the real-time task allocation requirements in dynamic environments. In this paper, CBBA is improved to further solve the above problems. The contributions of the paper are three-folds: firstly, based on the existing task selection and conflict mediation in CBBA algorithm, a new dynamic task generation process is proposed. Through the dynamic task generation, not only the timing constraints between tasks can be guaranteed, but also the complex tasks that need to be accomplished by multiple UAVs can be divided into multiple sub-tasks that only need one UAV to complete.

Secondly, the new method of reconstructing partial path is developed. By re-participating some low-income tasks in task allocation, not only the optimal result of the allocation is achieved, but also the real-time performance of the allocation process is guaranteed. In addition, in order to ensure the real-time performance of the algorithm, the concept of asynchronous task allocation is introduced in this paper. By assigning tasks to UAVs at different time points and mediating only one task at each communication time, the time and communication load of the algorithm are greatly reduced under the premise of ensuring the performance of the algorithm.

Thirdly, we constructed a simulation platform and performed dynamic task assignment experiments, it is verified that the algorithm can achieve functions mentioned above. As a contribution to the research community, this platform can be used by other academic researchers for validation testing purposes.

## Figures and Tables

**Table 1:** Decision rules for agent $V_{j}$ (receiver) upon receiving message from agent $V_{i}$ (sender).

$x_{i}^{k}$ is	$x_{j}^{k}$ is	Action of Agent $V_{j}$
i	j	if $y_{i}^{k}>y_{j}^{k}$, update
	i	update
	m∉{i,k}	if $s_{i}^{m}>s_{j}^{m}$ or $y_{i}^{k}>y_{j}^{k}$, update
	∅	update
j	j	leave
	i	reset
	m∉{i,k}	if $s_{i}^{m}>s_{j}^{m}$, reset
	∅	leave
m∉{i,k}	j	if $s_{i}^{m}>s_{j}^{m}$ or $y_{i}^{k}>y_{j}^{k}$, update
	i	if $s_{i}^{m}>s_{j}^{m}$, update; else, reset
	m	if $s_{i}^{m}>s_{j}^{m}$, update
	n∉{i,k,m}	if $s_{i}^{m}>s_{j}^{m}$ and $s_{i}^{n}>s_{j}^{n}$, update
		if $s_{i}^{m}>s_{j}^{m}$ and $y_{i}^{k}>y_{j}^{k}$, update
		if $s_{i}^{n}>s_{j}^{n}$ and $s_{j}^{m}>s_{i}^{m}$, reset
	∅	if $s_{i}^{m}>s_{j}^{m}$, reset
∅	j	leave
	i	update
	m∉{i,k}	if $s_{i}^{m}>s_{j}^{m}$, update
	∅	leave
